# C-A-S-H Gel and Pore Structure Characteristics of Alkali-Activated Red Mud–Iron Tailings Cementitious Mortar

**DOI:** 10.3390/ma15010112

**Published:** 2021-12-24

**Authors:** Chao Li, Na Zhang, Jiancong Zhang, Shuai Song, Yihe Zhang

**Affiliations:** Beijing Key Laboratory of Materials, Utilization of Nonmetallic Minerals and Solid Wastes, National Laboratory of Mineral Materials, School of Materials Science and Technology, China University of Geosciences, Beijing 100083, China; SleeQaQ@126.com (C.L.); zjc1321695230@126.com (J.Z.); 18600771568@163.com (S.S.)

**Keywords:** strength development verification, reaction product, SEM-EDS analysis, relative bridge oxygen, embodied carbon

## Abstract

Red mud and iron tailings are representative solid wastes in China, which have caused serious environmental pollution and potential harmful risk to people. Based on the alkali characteristic of Bayer red mud and natural fine-grained feature of iron tailings, these two solid wastes were used as raw materials to prepare alkali-activated cementitious mortar (AACM). The microstructure of C-A-S-H gel, pore structure characteristics, environmental impact and economic potential of this AACM were investigated. The results show that C-A-S-H gel was mainly composed of SiQ^3^ structure in the 28-day cured AACM. The relative content of SiQ^4^ structure increased while that of SiQ^2^ structure decreased as the hydration time advanced from 7 to 28 days, resulting in the increase of relative bridge oxygen value by 11.02%. The pores in the AACM sample accounted for 6.73% of the total volume, and these pores were not connected. The pore distribution was relatively uniform, which supported the good development of mechanical strength for AACM. This research elucidates the formation mechanism of C-A-S-H gels in the Bayer red mud–iron tailings-based AACM. In addition, the lower embodied carbon and material cost demonstrate that the prepared AACM has great environmental benefit and certain economic potential.

## 1. Introduction

Red mud is a kind of industrial solid waste produced in the process of extracting alumina from bauxite. Its main chemical components are SiO_2_, Al_2_O_3_, CaO, Fe_2_O_3_, Na_2_O, and TiO_2_. Every one ton of alumina is produced with the discharge of 1.0–1.8 tons of red mud. Because red mud contains high alkali content, its piling up in large quantities can seriously cause environmental pollution [1,2], such as land alkalinization and groundwater pollution, which will inevitably affect people’s health. As a major producer of alumina, China emits millions of tons of red mud each year [3]. Most red mud is stored in open air without any effective use. According to statistics, China’s alumina production in 2018 is about 69 million tons [4]. If the red mud produced by per ton of alumina is calculated as 1.45 tons, China’s red mud production in 2018 is about 100 million tons. Just as other solid wastes need to be disposed with detoxification and solidification, such as municipal solid waste incineration fly ash [5], the hazardous components of red mud also need to be disposed of, and the heavy metals need to be solidified and stabilized before it can be used. With the problem of red mud stacking and its pollution to the environment becoming more and more serious, it is extremely urgent to utilize red mud to the maximum extent.

As an industrial solid waste generated in the process of iron ore beneficiation, iron tailings discharge more than 10 billion tons of tailings and waste rocks every year in the world [6]. Over the past thirty years, with the rapid development of China’s iron and steel industry, iron tailing has become one of the main industrial solid wastes in China. By 2015, China has produced 5 billion tons of iron tailings, with a yearly discharge of more than 600 million tons, accounting for 1/3 of the total tailings reserves [7,8]. The massive accumulation of iron tailings leads to the waste of land resources, which greatly increases the possibility of environmental pollution [9,10]. The residual ions of flotation agent in iron tailings enter the surrounding fields and rivers with rainwater, which will cause serious pollution to the environment. Some harmless components in the tailings occur, i.e., differentiation, fragmentation, and various chemical reactions in nature, and the soluble ions generated will pollute the environment to some extent. Therefore, the resource utilization of iron tailings has been widely concerned by the whole society.

Alkali-activated cement (AAC) is a kind of inorganic cementitious material with three-dimensional net-like structure composed of AlO_4_ and SiO_4_ tetrahedral structure units. This material has excellent mechanical properties and performances of acid and alkaline resistance, fire resistance, and high temperature resistance [11]. It can use silica–alumina-based mineral waste and construction waste as raw materials. Because AAC is a new cementitious material with promising application prospect, it has been used far and wide in the field of building materials. Over the past twenty years, there has been much research on AAC using red mud or iron tailings as raw materials. Liu et al. [12] used Bayer red mud and coal metakaolin as raw materials, NaOH and sodium silicate as alkali activators to prepare AAC with 28-day compressive strength up to 56.2 MPa by adjusting Na/Al molar ratio to be 1.0. Their research shows that the increase of Na/Al molar ratio in a certain range is conducive to the development of pore structure, and finally leads to the improvement of mechanical properties. However, the continuous increase of Na/Al molar ratio will lead to the alkalinity of AAC and destroy the microstructure of AAC. Koshy et al. [13] used red mud, coal gangue and fly ash after silicate activation treatment as raw materials to prepare AAC. Their study found that the ternary mixture of coal gangue, fly ash and red mud had a higher strength than the binary mixture of coal gangue and red mud at a lower curing temperature. Duan et al. [14] used iron tailings and fly ash as raw materials to prepare AAC. The effects of temperature and times of thermal cycling, and the proportion of iron tailings on the properties of AAC were studied. Their results show that addition of iron tailings optimized the surface Vickers-hardness of the AAC, and the thermal resistance of AAC was significantly improved with the replacement level of iron tailings less than 30%, and replacing fly ash with 20% iron tailings would obtain a much denser microstructure because of the reduction of porosity and micro-crackings. Defáveri et al. [15] used iron tailings and glass wool residue as raw materials to prepare AAC with compressive strength higher than 100 MPa and flexural strength higher than 20 MPa. They found that C-S-H gel existed in the iron tailings-based AAC. Moreover, using iron tailings as main raw material and metakaolin as correction raw material, Chen et al. [16] prepared AAC with 28-day compressive strength up to 59.0 MPa by alkali activation. By conducting orthogonal experiments, they concluded that Si/Al ratio of raw materials had the greatest influence on early strength of iron tailings–metakaolin-based AAC among all factors, and liquid-solid ratio had the greatest influence on the final strength. They found that geological polymerization and hydration reactions were carried out simultaneously in the iron tailings–metakaolin-based AAC, and some mineral crystalline phases gradually changed into amorphous structure. The main phase of iron tailings–metakaolin-based AAC was amorphous silicoaluminate, semi-crystalline CSH (I) and α-C_2_SH, and the gelatinous substance cemented the fine particles together to form a compact gel body, which made the AAC have a high compressive strength. Besides, Wang et al. [17] also found that the Si/Al ratio had a great influence on the properties of geopolymer, and the varied Si/Al ratio resulted in different structures, properties, and efflorescence degree of the fly ash-based AAC.

Although there have been many studies on the preparation of AAC from red mud or iron tailings, there are still few studies on the preparation of AAC by using both these two solid wastes as raw materials. Most of iron tailings are ground into powder as silicon–aluminum-based precursors to participate in the AAC reaction. There are few studies on preparation of AAC from unground iron tailings. Our research group has made a new attempt to produce red mud–iron tailing-based alkali-activated mortar and investigated the mechanical properties and environmental stability performance including resistances to acid, alkali, and sulfate attacks as well as freeze-thaw cycling of the red mud–iron tailing-based alkali-activated mortar [18]. It was found that the best performance was attained for the alkali-activated mortar composed of 16% Bayer red mud powder and 75% iron tailing aggregate. Based on the previous study, the present work is a further deep investigation on the C-A-S-H gel formation mechanism and pore structure characteristics of Bayer red mud–iron tailings-based alkali-activated cementitious mortar (AACM). In this research, Fourier transform infrared spectroscopy (FT-IR), ^29^Si nuclear magnetic resonance (NMR), and scanning electron microscopy combined with energy dispersive spectroscopy (SEM-EDS) were applied to obtain useful information on the phase identification, SiO_4_ polymerization degree, micro-morphology, and element composition of C-A-S-H gels in the Bayer red mud–iron tailings-based AACM. Computed tomography (X-CT) technique was employed to analyze the pore structure of this AACM. Moreover, environmental impact and economic potential of this AACM were evaluated. The paper is hoped to provide a corresponding theoretical support for the large-scale industrial utilization of iron tailings and red mud in the production of AAC.

## 2. Materials and Methods

### 2.1. Materials

**Figure 1:**
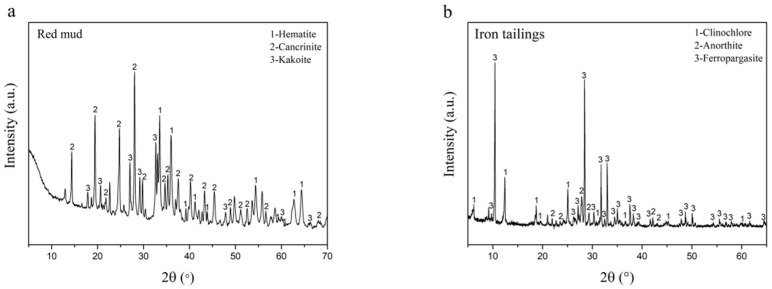
XRD patterns of Bayer red mud (**a**) and iron tailings (**b**).

The main raw materials for preparing AACM in this study are Bayer red mud, blast furnace slag powder, and iron tailings. Besides, a small amount of 52.5 Portland cement was added to provide calcium-based precursor. Table 1 shows the chemical composition and origin information of the relevant raw materials used in this work. As the most important raw materials, Figure 1 shows XRD patterns of Bayer red mud and iron tailings. It presents that the main mineralogical phases of Bayer red mud are cancrinite, katoite, and hematite. The main minerals composed in the iron tailings are ferropargasite, clinochlore, and anorthite.

**Figure 2:**
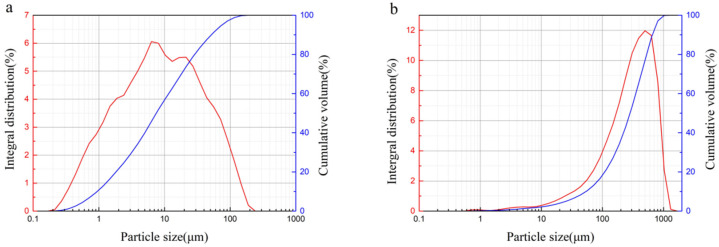
Laser particle size distribution of Bayer red mud (**a**) and fine iron tailings (**b**).

Coarse and fine iron tailings with different particle size were used in this research. The particle size of coarse iron tailings is distributed within 3–5 mm. Figure 2 shows laser particle size distributions of Bayer red mud and fine iron tailings. As can be seen from Figure 2a, the particle size of red mud is relatively small, and it is mainly distributed within the range of 0.3–120 µm. The particle size of fine iron tailings is relatively larger than that of red mud, and its particle size is mainly distributed within the range of 21–1028 µm.

### 2.2. Preparation of AACM

**Figure 3:**
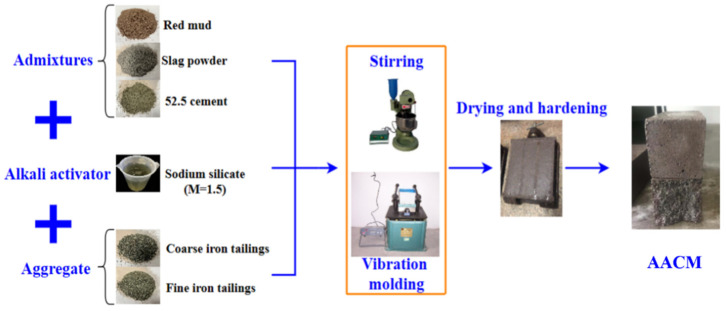
Preparation flow chart of AACM.

The AACM was prepared by mixing the Bayer red mud, iron tailings, blast furnace slag powder, 52.5 Portland cement, alkali activator and water together. The mass proportions of Bayer red mud, iron tailings, blast furnace slag powder, and 52.5 Portland cement were 16%, 75%, 7.5%, and 1.5%, respectively. Sodium silicate was used as alkali activator to participate in the geopolymerization, and the modulus in this work was set to 1.5. In addition, the mass ratio of coarse and fine iron tailings as aggregates was 3:2, and the ratio of water to binder was 0.63. The preparation flow chart of AACM is shown in Figure 3. Firstly, admixtures were added to the blender, then turned on the blender, poured into the sodium silicate and water, and stirred for 30 s at 62 rpm. Secondly, coarse and fine iron tailings were added to the blender and stirred for another 30 s at 62 rpm. Finally, stirring for 90 s at 125 rpm to complete the mixing of raw materials. The slurry was then poured into a 40 × 40 × 160 mm mortar mold and vibrated for 2 min to remove the entrapped air from the mixture. To prevent evaporation, the fresh mortar should be covered with plastic wrap. After 24 h, they were demolded at room temperature and then cured in a constant temperature and humidity curing box with a temperature of 20 ± 2 °C and a humidity of 90%.

### 2.3. Testing and Characterization

X-ray powder diffractometer (D8 ADVANCE, Bruker Corporation, Massachusetts, America) is used for mineralogical phase characterization of Bayer red mud and iron tailings. The target source is equipped with Cu target and Co target. The diffraction angle range (2θ) is 5–70° with scanning rate of 6°/min. The test temperature is 25 °C, the tube voltage is 20–60 kV, and the tube current is 10–60 mA. The samples were analyzed by JADE 6.0 software. X-ray fluorescence spectrometer (ARLAdvantX Intellipower TM3600, Thermo Fisher Scientific, Waltham, MA, USA) is used to analyze chemical composition of Bayer red mud, iron tailings, blast furnace slag powder, and 52.5 Portland cement. Laser particle size distribution instrument (Bettersize2000, Dandong Baxter Instrument Co., Ltd., Dandong, China) is used to test the particle size distribution of Bayer red mud and fine iron tailings.

Compressive strength and flexural strength of AACM specimens were tested according to Test Method for Strength of Cement Mortar (ISO method) (GB/T17671-1999) [19] when the curing time reached 3, 7 and 28 days. The flexural strength and compressive strength are two important mechanical performance indexes of AAC material. In this study, the devices used to test the flexural strength and compressive strength are motorized bending tester (KZJ-500, Shenyang Great Wall Electromechanical Equipment Factory, Shenyang, China) and electro-hydraulic servo universal testing machine (WAW-2000E, Jinan Koohei Test Machine Co., Ltd., Jinan, China), respectively.

Characterization tests including SEM-EDS, FT-IR, NMR, and X-CT techniques were performed on the AACM specimens cured for 7 and 28 days. Before these characterization tests, it is required to carry out a series of treatments for the samples. After curing for 7 and 28 days, the AACM samples were immersed in anhydrous ethanol for 72 h to stop the hydration process, and then the samples were dried in a vacuum drying oven at 60 °C for 24 h. Finally, some of the samples were ground into powder by electromagnetic sample grinder for FT-IR and NMR analysis, and the other samples without grinding were directly taken for SEM and X-CT analysis.

Field emission scanning electron microscope (SEM, SU8020, Hitachi Ltd., Tokyo, Japan) is used to observe the micromorphology of AACM specimens. Before taking SEM image, the AACM samples need to be treated with golden sputtering.

Infrared spectrometer (Nicolet IS10, Thermo Nicolet Corporation, Madison, GA, USA) is used for FT-IR test. Through analyzing the infrared spectrum of sample, the absorption band can be used to determine the functional groups to determine the corresponding chemicals and alkali reaction products of AACM.

The equipment used for solid nuclear magnetic resonance testing is 600 M solid nuclear magnetic resonance instrument (Agilent 600M, Agilent Technologies Inc., Santa Clara, CA, USA). The amount of powdered sample used for NMR testing should be more than 0.5 g. After completion of the NMR test, MestReNova software was used to analyze and process the test data. Through analysis of ^29^Si NMR spectrum of AACM, the SiO_4_ structure unit and relative bridge oxygen (RBO) value of hydration product can be obtained, and then the change rule of SiO_4_ polymerization degree of AACM with hydration time can be understood.

Three dimensional CT scanner (NanoVolex 4000, Tianjin Sanying Precision Instrument Co., Ltd., Tianjin, China) is used to test the pore structure of AACM. One picture with a size of 1920 × 1536 was collected at an interval of 0.25°. We improved the resolution of 2880 images by rotating the sample twice during the test. Other experimental parameters are presented in Table 2. The reconstruction software Voxel Studio Recon was used to carry out the algorithm reconstruction, image correction, and processing of the scanned data after the completion of the test. Then, Volume Graphics Studio Max, FEI Avizo, SYPI-Sore and other software were used to carry out image display, measurement, digital core analysis, and other processing on the reconstructed data of the sample. The sample also needs to be processed before the test. After curing for 7 days, the AACM sample with size of 40 × 40 × 160 mm was cut into a cube specimen with size of 30 × 30 × 30 mm. The specimen was immersed in anhydrous ethanol for 72 h to stop the hydration process, and then dried in a vacuum oven at 60 °C for 24 h.

## 3. Results and Discussion

### 3.1. Strength Development of the AACM

**Figure 4:**
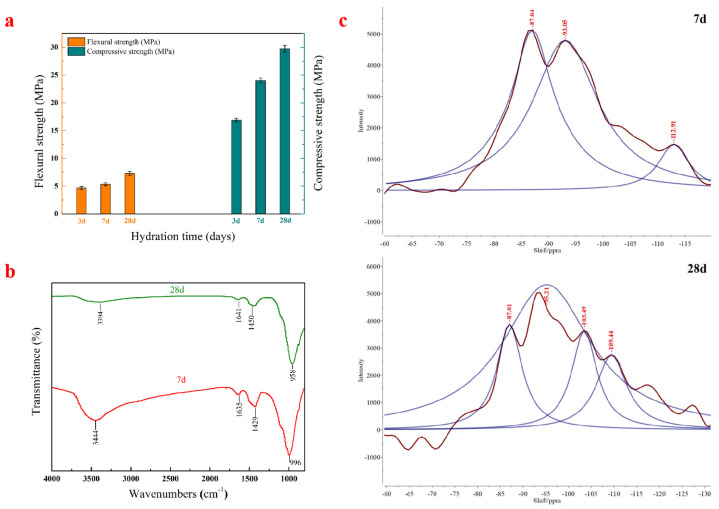
Flexural and compressive strengths (**a**), FI-IR spectra (**b**), and ^29^Si MAS NMR spectra (**c**) of AACM hydrated for 7 and 28 days.

The flexural and compressive strengths of AACM with hydration time are shown in Figure 4a. With the increase of hydration time, the flexural strength and compressive strength of AACM also increase. At 3 days of hydration, the flexural strength and compressive strength of the specimen reached values of 4.68 and 16.88 MPa, respectively, and then they reached 7.28 and 29.74 MPa after 28 days of hydration. Compared with our previous work [18], the strength development of this AACM is slower than that of sample AB presented in the preliminary work. As the origins of red mud, iron tailings, and blast furnace slag have changed in this work, the different chemical and mineralogical compositions of red mud and iron tailings result in the strength difference. However, the 28-day flexural and compressive strengths are similar, which can basically attain 7 and 30 MPa. It is thought that the produced AACM is appropriate for road repair because of its high early strength. Furthermore, this AACM is prepared with large proportions of solid wastes (red mud, iron tailings, and blast furnace slag) as raw material, which can not only relieve the accumulation pressure of solid wastes, but also save cost and potentially produce huge economic benefits.

### 3.2. C-A-S-H Gels Formation in the AACM

#### 3.2.1. FT-IR Analysis

Figure 4b shows FT-IR spectra of AACM specimens hydrated for 7 and 28 days. The spectral bands of specimens hydrated for 7 days and 28 days are similar. The absorption peaks with wavenumber at 1429 and 996 cm^−1^ are the characteristic spectral bands of C-A-S-H gel [18,20,21]. It proves that the reaction product of this AACM is mainly C-A-S-H gel. The spectral band at 996 cm^−1^ is put down to the anti-symmetric stretching vibration of Si-O-Si(Al) in the Si(Al)O_4_ tetrahedral structure of C-A-S-H gel. The wide absorption spectral band at 3444 cm^−1^ represents stretching vibration of Al-OH in the cancrinite of red mud. The peak decreases when the specimen hydrates for 28 days, which indicates that Al-OH in the red mud participates in the polymerization reaction to form more C-A-S-H gels. The absorption spectral band at 1635 cm^−1^ represents H-O-H bending vibration for interlayer water [22,23]. As the hydration time increases, free water participates in the hydration process and gradually changes into crystal water.

#### 3.2.2. ^29^Si NMR Analysis

Figure 4c shows ^29^Si NMR spectra of AACM specimens hydrated for 7 and 28 days. ^29^Si NMR can provide information about the relative amount of silicon atoms in different Q^n^ (mAl) (n = 0–4) tetrahedra environments, where n represents the number of bridge oxygen between SiO_4_ tetrahedrons, and the number of aluminum atoms around SiO_4_ tetrahedron is represented by m [24]. Through the analysis of ^29^Si NMR spectrum, the polymerization degree of aluminum–silicate chain can be obtained. Therefore, the formula for calculating the relative bridge oxygen (RBO) number that can effectively evaluate the polymerization degree of SiO_4_ is summarized [25]):

(1) $$\mathrm{RBO}=\frac{1}{4}\left(1\times \frac{Q^{1}}{\sum Q^{n}}+2\times \frac{Q^{2}}{\sum Q^{n}}+3\times \frac{Q^{3}}{\sum Q^{n}}+4\times \frac{Q^{4}}{\sum Q^{n}}\right)=\frac{1}{4}\times \frac{\sum n\cdot Q^{n}}{\sum Q^{n}}$$

Q^n^: Relative area of the formant.

The research of Puertas et al. [26] states that the peaks at −77 to −82 ppm corresponds to SiQ^1^ unit, and the peaks near −85 ppm corresponds to SiQ^2^ unit. The peak near −82 to −84 ppm is related to SiQ^2^ (1Al)/SiQ^2^ (0Al), which is mainly due to the signal moving from 3–5 ppm to more positive values by replacing Si with Al. Besides, the chemical shift from −92 to −100 ppm is associated with SiQ^3^ unit, and the peak near −88 ppm to −91 ppm is related to SiQ^3^ (2Al)/SiQ^3^ (1Al). The peaks at −103 to −115 ppm corresponds to SiQ^4^ unit. According to the data in Table 3, it can be known that the C-A-S-H gels in the AACM specimen hydrated for 7 days are mainly composed of SiQ^3^ and SiQ^2^ units, while they mainly contain SiQ^3^ structure at 28 days. The RBO value of the 28-day sample increases by 11.02% compared with that of the 7-day specimen. It indicates that the extension of the hydration time leads to the increase of SiO_4_ tetrahedron polymerization degree for the C-A-S-H gels. After hydrated for 28 days, the structure of C-A-S-H gels become more complicated with SiQ^4^ structure increased and SiQ^2^ structure decreased.

#### 3.2.3. SEM-EDS Analysis

**Figure 5:**
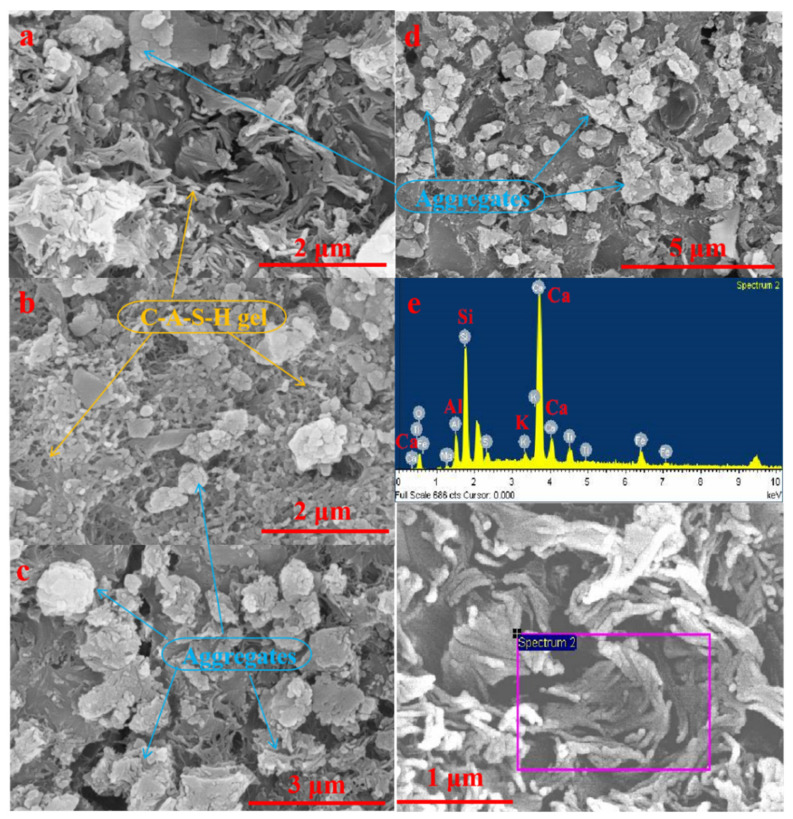
SEM morphology (**a**–**d**) and EDS analysis (**e**) of C-A-S-H gels in the AACM after 7 days of hydration.

**Figure 6:**
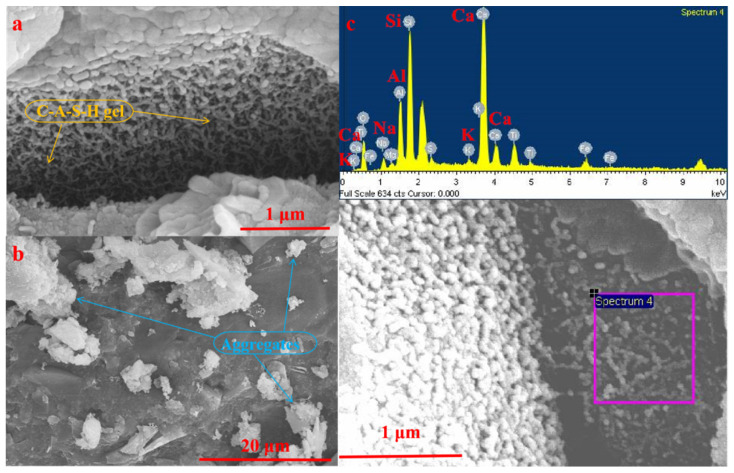
SEM morphology (**a**,**b**) and EDS analysis (**c**) of C-A-S-H gels in the AACM after 28 days of hydration.

Figure 5 and Figure 6 show the SEM morphologies of AACM specimens hydrated for 7 and 28 days, respectively. The SEM analysis results show that the cementitious substance of AACM in this work is mainly reticular gel. In addition, it can be seen that the iron tailings particles are embedded in the gel matrix and bonded by the gels, indicating that the iron tailings aggregates are closely connected and cemented by the reticular gels.

The results of EDS analysis on the reticular gels occurring in the AACM specimens hydrated for 7 and 28 days are shown in Table 4. As the reticular gels contain Ca, Si and Al elements, it can be firmly determined that the reaction product of this AACM is C-A-S-H gel. This result is in accordance with the above FT-IR analysis result and the observation reported by Ding et al. [27]. In addition, according to the data in Table 4, it can be known that with the prolongation of hydration time, the content of Al and Na+K in the C-A-S-H gel at 28 days increases significantly compared with that at 7 days, and the content of Si and Ca decreases correspondingly. Owing to the substitution of Si by Al in the C-A-S-H gel, the number of AlO_4_ connected with SiO_4_ increases with the extension of hydration time, and on this basis, the number of monovalent cations (such as Na^+^ and K^+^) that attracted for solidification due to the charge vacancy also increases correspondingly. The efflorescence degree can be obviously reduced by the dense matrix and AlO_4_ structures of AAC [17]. In our previous work reported by Song et al. [18], the main products generated in the 28-days cured red mud–iron tailings-based AACM are C-A-S-H gels and calcium aluminum iron silicate hydroxide (Ca_3_AlFe(SiO_4_)(OH)_8_). However, except the main product of C-A-S-H gel, calcium aluminum iron silicate hydroxide is not found in the present work. It is noticed that the Bayer red mud used in our previous work contains 46.61% Fe_2_O_3_, while the red mud used in the present study only contains 7.67% Fe_2_O_3_. Although cancrinite is composed in the present red mud, the much lower content of Fe_2_O_3_ is insufficient to support the replacement of Al by Fe for the transformation of cancrinite to form Ca_3_AlFe(SiO_4_)(OH)_8_.

#### 3.2.4. Formation Mechanism of C-A-S-H Gel in the AACM

**Figure 7:**
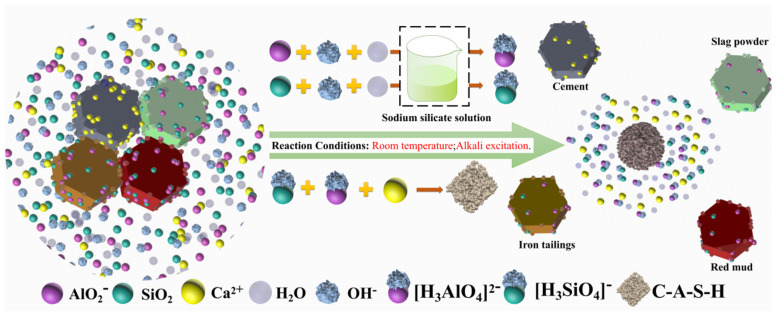
Schematic diagram of the formation of C-A-S-H gels in the AACM.

Red mud and blast furnace slag powder adopted in this study are silica-alumina precursors, which are the key materials to prepare AACM. 52.5 Portland cement acts as the calcium-silica precursor to catalyze the geopolymerization process. The alkali-activated reaction product of this AACM is mainly amorphous C-A-S-H gel, which plays an important part in facilitating the densification of the AACM structure [28,29,30]. The formation process of C-A-S-H gel in this AACM can be expressed as the following, the schematic diagram of which is shown in Figure 7.

SiO_2_ + OH^−^ + H_2_O → [H_3_SiO_4_]^−^ (2)

AlO_2_^−^ + OH^−^ + H_2_O → [H_3_AlO_4_]^2−^ (3)

[H_3_SiO_4_]^−^ + [H_3_AlO_4_]^2−^ + Ca^2+^ → C-A-S-H (4)

**Figure 8:**
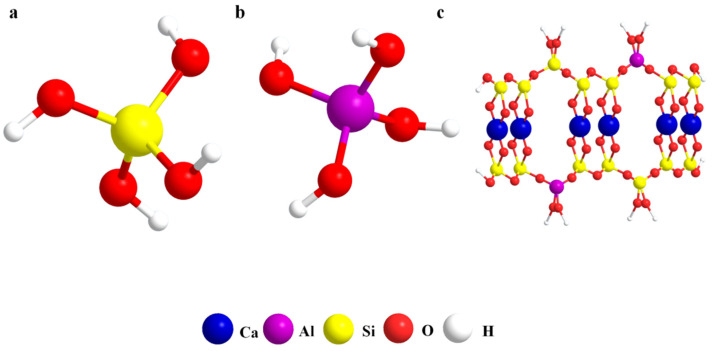
Structural model. ((**a**): [H_3_SiO_4_]^−^, (**b**): [H_3_AlO_4_]^2−^, and (**c**): C-A-S-H gel).

The active siliceous substances in red mud, blast furnace slag powder, and iron tailings are uniformly represented by SiO_2_. AlO_2_^−^ represents the active aluminum substances dissolved from red mud, blast furnace slag powder, and iron tailings. When the alkali-activated reaction and hydration begin, the 52.5 Portland cement and sodium silicate solution quickly release ions of Ca^2+^, OH^−^, [H_3_SiO_4_]^−^. Under the activation provided by the 52.5 Portland cement and alkali activator, [H_3_SiO_4_]^−^ and [H_3_AlO_4_]^2^^−^ ions are continuously dissolved from the active SiO_2_ and Al_2_O_3_ in the red mud, blast furnace slag powder, and the surface of iron tailings. In this blended system, the amorphous C-A-S-H gels are formed by the reaction of [H_3_SiO_4_]^−^ and [H_3_AlO_4_]^2^^−^ ions with Ca^2+^, and their structural model is shown in Figure 8 [31,32,33]. During the hydration reaction, the dissolved K^+^ and Na^+^ in this system can combine with [SiO_4_]^4^^−^ and [AlO_4_]^5^^−^ to form transitional substances. As the hydration time increases, the hydration reaction continues, and a large amount of dissolved Na^+^ and K^+^ could be stabilized in the amorphous gel. Na^+^ and K^+^ play the roles of catalysis and participation in charge balance in this reaction [34,35]. As the main alkali-activated reaction product, amorphous C-A-S-H gels are not only the main reason for the strength development of AACM, but also conducive to the refinement of the pore size, which makes a dense structure of AACM.

### 3.3. Pore Structure Characteristics of the AACM

**Figure 9:**
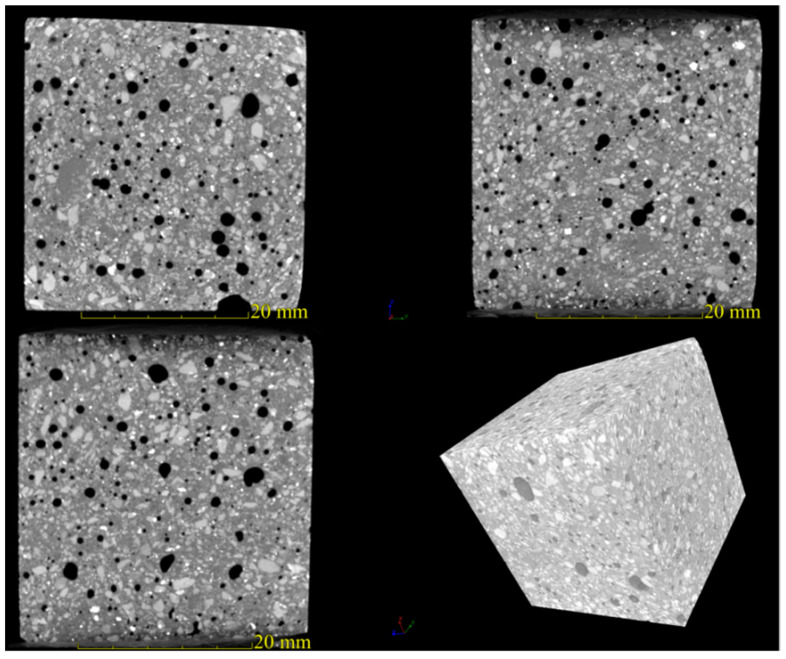
Four views of AACM specimen.

**Figure 10:**
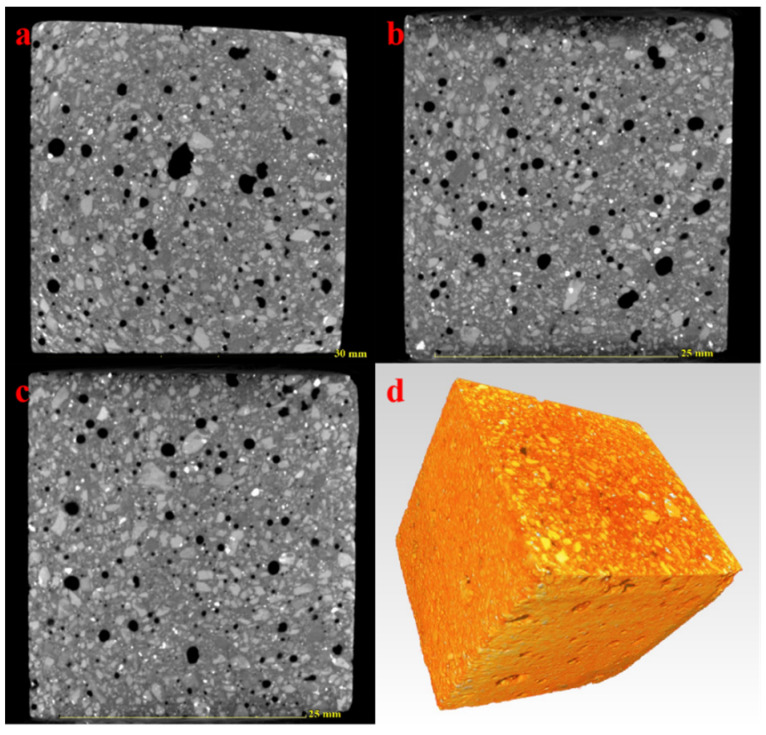
XY, XZ, YZ directional slices (**a**–**c**) and 3D scanning image of AACM specimen (**d**).

In order to observe the large pore structure of AACM, NanoVolex 4000 series resolution industrial CT was adopted in this study to scan the hardened AACM specimen that have hydrated for 7 days. Two-dimensional (2D) and three-dimensional (3D) imaging from different directions were applied to scan the sample. The four views of the AACM sample are shown in Figure 9. The distribution of pores and other components can be clearly seen from Figure 9. The black area represents pores, the white light part represents metallic minerals, and the gray part represents aggregates and other matrix materials. Moreover, by scanning the sample slices in XY, XZ, and YZ directions, we can further explore whether the pores of AACM are evenly distributed in different directions. According to Figure 10, the pore distribution of AACM is evenly distributed in the above three directions. It can be seen intuitively that the size of pores in the directions of XY and XZ are larger than those in YZ direction. In general, the presence of large pores is not conducive to the strength development of materials.

**Figure 11:**
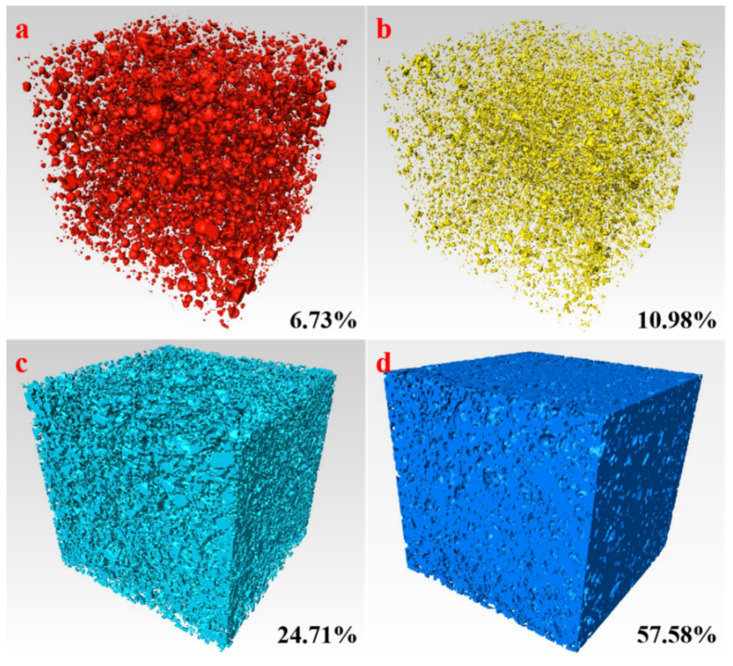
Three dimensional distribution of different components of AACM specimen. ((**a**): pore, (**b**): mineral, (**c**): aggregate particle, and (**d**): other matrix material).

Digital core analysis and threshold segmentation are used for quantitative analysis of the different components of the material in order to understand the pore structure of AACM at a greater distance. The principles and applications of digital core analysis and threshold segmentation have been described in detail in the literature [36,37]. The computer software FEI AVIZO is used to threshold the images of AACM. Figure 11 shows the 3D distribution of pore, mineral, aggregate particle, and other matrix material obtained by processing data. The red substances in Figure 11a represent pores in the sample, and it accounts for 6.73% of the total volume of AACM specimen. According to Figure 11a, the different pores of the prepared AACM are uniformly distributed, and the pores with small size are in the majority, which is beneficial to improve the mechanical properties of AACM. Furthermore, the yellow, cyan, and blue substances in Figure 11b–d represent minerals, aggregate particles, and other matrix materials, respectively, and they account for 10.98%, 24.71%, and 57.58% of the total volume, respectively. In the total volume of the specimen, the proportions of aggregate particles and other matrix materials have reached 82.29%, which guarantee the strength requirements of AACM.

**Figure 12:**
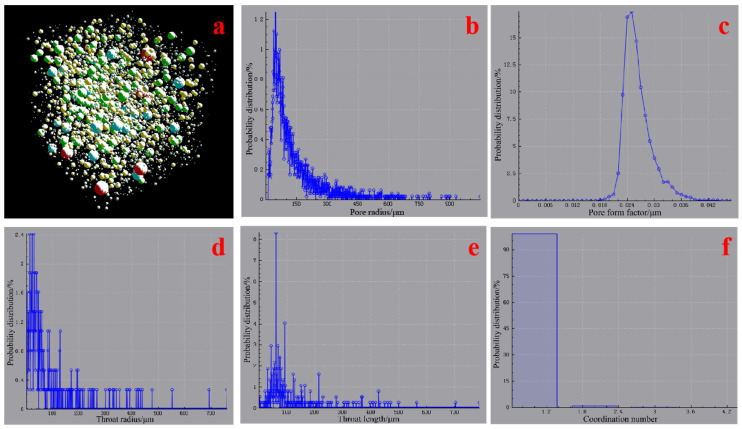
Characterization parameter analysis diagram of AACM specimen. ((**a**): ball and stick model, (**b**): pore radius, (**c**): pore form factor, (**d**): throat radius, (**e**): throat length, and (**f**): coordination number).

In order to better understand the pore structure of the AACM sample, it is necessary to use the maximum sphere algorithm to process the pore data obtained from digital core analysis and construct the pore network model. The principles of the maximum sphere algorithm have been described in detail in the literature [36,38]. The parameters of the sample such as pore throat size, coordination number, and shape factor can be obtained by mathematical statistics. The pore throat network model constructed in this study and the related characterization parameters obtained are shown in Figure 12. According to Figure 12b,d, the pores and throats of the sample have skewed distributions. Large pores and throats are relatively few. The pore radius and throat radius are mainly distributed in 50–100 and 20–60 µm, respectively. Figure 12c shows that the pore form factor has a normal distribution, and the main distribution values are 0.02–0.032. According to the relevant introduction of the form factor in the literature [36], it can be concluded that the shape of most of pores and throats in this study area is roughly triangular and irregular. According to Figure 12e, the distribution of throat length in the study area is mainly 60 µm, indicating that one part of the throat has a high passability. The coordination number is the number of throats connected by a pore. The larger the coordination number, the more the number of throats coordinated with it, and the better the connectivity of the material. As shown in Figure 12f, the maximum coordination number in the study area of AACM sample is 4, and the average coordination number is 0. It indicates that the pores in the AACM are not connected.

### 3.4. Environmental Impact and Economic Potential of the AACM

In this study, embodied carbon, an important sustainability parameter, is used to assess the environmental impact of AACM. The embodied carbon of raw materials are calculated based on the cradle-to-gate system, and the calculation method of embodied carbon of AACM comes from the literature [39]. The embodied carbon of the raw materials comes from the statistics of Hammond and Jones [40], and the specific data are presented in Table 5. Among them, the embodied carbon of red mud has not been found, but its embodied carbon is estimated to be almost the same as that of solid waste such as fly ash; thus, it is considered to be less than 0.1 when calculating the total embodied carbon of AACM. In this work, as the 28-day compressive strength of this AACM is about 30 MPa, C30 concrete is selected as the reference standard when studying the environmental impact of AACM [41].

**Figure 13:**
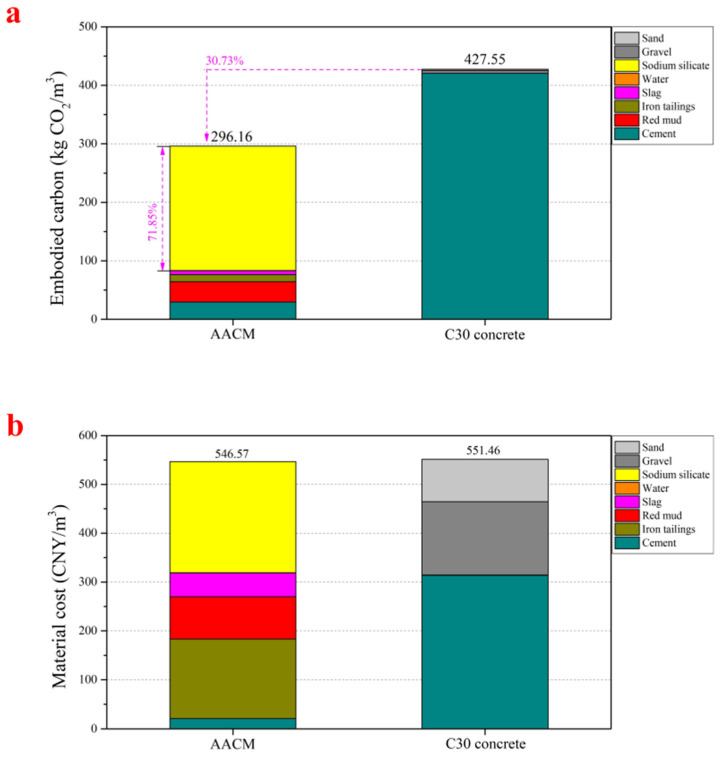
Embodied carbon and material cost of AACM. ((**a**): embodied carbon, (**b**): material cost).

The comparison chart of the total embodied carbon of AACM and C30 concrete is shown in Figure 13a. The total embodied carbon of AACM is 30.73% lower than that of C30 concrete. Furthermore, in the total embodied carbon of AACM, the embodied carbon of alkali activator accounted for a large proportion of 71.85%, and the embodied carbon of other raw materials accounted for a small proportion. Therefore, it demonstrates that using red mud, blast furnace slag, and iron tailings to prepare AACM is extremely environmental friendly.

Figure 13b shows material cost comparison between AACM and C30 concrete. It is known that the material cost of alkali-activated cement is generally higher than that of the traditional cement concrete at an equal strength level, mainly owning to the high cost of alkali activator. The material cost of AACM is calculated to be 546 CNY/m^3^. Although the cost of alkali activator accounts for 41.6% of the total material cost, this AACM attains lower materials cost than the reference C30 concrete. It suggests that using iron tailings as aggregates, red mud, blast furnace slag, and a small amount of Portland cement as precursors to prepare AACM has a certain economic potential.

## 4. Conclusions

In this work, the C-A-S-H gel formation mechanism, pore structure characteristics, environmental impact and economic potential of Bayer red mud–iron tailings-based AACM were further investigated based on our published work [18]. In order to verify the strength development and the general reaction product of Bayer red mud–iron tailings-based AACM, the origins of red mud, iron tailings, and blast furnace slag have changed in the present work. The flexural and compressive strengths of the AACM prepared in this work reach 7.28 and 29.74 MPa after 28 days of hydration, which are basically close to the 28-day strength reported in the literature [18]. The early-strength characteristic endows the red mud–iron tailings-based AACM potentially being applied as road repair materials.

FT-IR and SEM analysis results prove that the reaction product of this AACM is mainly C-A-S-H gel. ^29^Si NMR analysis results indicate that the C-A-S-H gels in the AACM specimen hydrated for 7 days are mainly composed of SiQ^3^ and SiQ^2^ units, while they mainly contain SiQ^3^ structure at 28 days. With the extension of the hydration time from 7 to 28 days, the RBO value increases by 11.02%, indicating a significant increase of SiO_4_ tetrahedron polymerization degree of C-A-S-H gels. After being hydrated for 28 days, the structure of C-A-S-H gels becomes more complicated with SiQ^4^ structure increased and SiQ^2^ structure decreased.

SEM observation shows that the iron tailings aggregates are closely connected and cemented by the reticular C-A-S-H gels. With the prolongation of hydration time from 7 to 28 days, the content of Al and Na+K in the C-A-S-H gel increases significantly, while the content of Si and Ca decreases correspondingly. Owing to the substitution of Si by Al in the C-A-S-H gel, the number of AlO_4_ connected with SiO_4_ increases with the extension of hydration time, and on this basis, the number of monovalent cations (such as Na^+^ and K^+^) that attracted for solidification due to the charge vacancy also increases.

The formation mechanism of this AACM is clarified. When the alkali-activated reaction and hydration begin, the 52.5 Portland cement and sodium silicate solution quickly release ions of Ca^2+^, OH^−^, [H_3_SiO_4_]^−^. Under the activation provided by the 52.5 Portland cement and alkali activator, [H_3_SiO_4_]^−^ and [H_3_AlO_4_]^2−^ ions are continuously dissolved from the active SiO_2_ and Al_2_O_3_ in the red mud, blast furnace slag powder, and the surface of iron tailings. In this blended system, the amorphous C-A-S-H gels are formed by the reaction of [H_3_SiO_4_]^−^ and [H_3_AlO_4_]^2−^ ions with Ca^2+^. In addition, as the hydration time increases, a large amount of dissolved Na^+^ and K^+^ can be stabilized in the amorphous C-A-S-H gels. Na^+^ and K^+^ play roles of catalysis and participation in charge balance in this reaction.

The data analysis results of X-CT show that the pore distribution of the AACM is relatively uniform, which is conducive to the development of mechanical strength. The pores in the AACM sample account for 6.73% of the total volume, and these pores are not connected. The minerals, aggregate particles, and other matrix materials account for 10.98%, 24.71%, and 57.58% of the total volume, respectively. The total embodied carbon and material cost of AACM are lower than those of the reference C30 concrete, demonstrating that the prepared AACM has great environmental benefit and certain economic potential.

The novelty of this work focuses on the explanation of the C-A-S-H gel formation mechanism in the red mud–iron tailings-based AACM and the clarification of its pore structure. It provides a theoretical support for the large-scale industrial utilization of iron tailings and red mud in the production of AACM.

## Figures and Tables

**Table 1:** Chemical composition and origin information of raw materials.

Chemical Constitution/%	Red Mud	Iron Tailings	Slag Powder	52.5 Portland Cement
Al_2_O_3_	24.66	17.87	17.3	6.05
SiO_2_	20.57	36.42	32.64	21.68
CaO	20.86	14.21	37.66	62.86
Na_2_O	16.16	0.94	0.5	0.2
Fe_2_O_3_	7.67	16.36	0.66	3.26
TiO_2_	6.17	2.42	0.72	0.32
SO_3_	1.08	-	1.63	2.61
K_2_O	0.62	1.08	0.76	1.09
MgO	0.39	6.41	7.42	1.51
Place of origin	Hejin, Shanxi Province of China	Laiyuan, Hebei Province of China	Hejin, Shanxi Province of China	Conch Cement Co. Ltd., Anhui Province of China

**Table 2:** Experimental parameters of X-CT test.

Sample	Resolution/μm	Voltage/kV	Current Flow/μA	Time of Exposure/s	Scanning Time/min
AACM	30.53	190	130	0.65	90

**Table 3:** RBO calculation results of C-A-S-H gels of AACM hydrated for 7 and 28 days.

Structural Unit	7 d		28 d	
	Chemical Shift (ppm)	Relative Area	Chemical Shift (ppm)	Relative Area
SiQ^2^	−87.0	79.4	−87.0	20.33
SiQ^3^	−93.0	100	−95.2	100
SiQ^4^	−112.9	14.4	−103.5	19.13
			−109.4	17.58
RBO	66.59%		77.61%	

**Table 4:** EDS analysis results of C-A-S-H gel (atomic percentage/%).

Chemical Element	7 d	28 d
O	41.30	51.35
Al	4.44	6.86
Si	17.71	15.17
Ca	26.85	17.85
Na+K	1.04	2.88

**Table 5:** Embodied carbon and market price of raw materials.

Material	Embodied Carbon (kg CO_2_/kg)	Market Price (CNY/Metric ton)
Iron tailings	0.00747	100
Red mud	<0.1	250
52.5 cement	0.912	630
Blast furnace slag	0.0416	300
Sodium silicate	2.28	850
Water	0.00034	4

## Data Availability

Date sharing is not applicable to this article.
